# Leveraging an epidemic–economic mathematical model to assess human responses to COVID-19 policies and disease progression

**DOI:** 10.1038/s41598-023-39723-0

**Published:** 2023-08-08

**Authors:** Wisdom S. Avusuglo, Nicola Bragazzi, Ali Asgary, James Orbinski, Jianhong Wu, Jude Dzevela Kong

**Affiliations:** 1 Africa-Canada Artificial Intelligence and Data Innovation Consortium (ACADIC), Laboratory for Industrial and Applied Mathematics, York University, Toronto, Canada; 2 Africa-Canada Artificial Intelligence and Data Innovation Consortium (ACADIC), The Advanced Disaster, Emergency and Rapid Response Program, York University, Toronto, Canada; 3 Africa-Canada Artificial Intelligence and Data Innovation Consortium (ACADIC), The Dahdaleh Institute for Global Health Research, York University, Toronto, Canada

**Keywords:** Computational biology and bioinformatics, Diseases, Health care, Risk factors, Mathematics and computing

## Abstract

It is imperative that resources are channelled towards programs that are efficient and cost effective in combating the spread of COVID-19, the disease caused by the Severe Acute Respiratory Syndrome Coronavirus 2 (SARS-CoV-2). This study proposed and analyzed control strategies for that purpose. We developed a mathematical disease model within an optimal control framework that allows us to investigate the best approach for curbing COVID-19 epidemic. We address the following research question: what is the role of community compliance as a measure for COVID-19 control? Analyzing the impact of community compliance of recommended guidelines by health authorities—examples, social distancing, face mask use, and sanitizing—coupled with efforts by health authorities in areas of vaccine provision and effective quarantine—showed that the best intervention in addition to implementing vaccination programs and effective quarantine measures, is the active incorporation of individuals’ collective behaviours, and that resources should also be directed towards community campaigns on the importance of face mask use, social distancing, and frequent sanitizing, and any other collective activities. We also demonstrated that collective behavioral response of individuals influences the disease dynamics; implying that recommended health policy should be contextualized.

## Introduction

The damage caused by infectious diseases since the dawn of mankind cannot be overemphasized—their destruction of health, economies and social life is crippling. They leave chaotic trails in their wake; some enter the human population and vanish progressively with time; others enter and stay: the need for the study of their transmission and control is ever-pressing. The world is saddled with another infectious disease—the novel “Coronavirus Disease 2019” (COVID-19). Numerous mathematical models that address its transmission and control have been studied in its wake (see, for example^1,2^ for extensive reviews on these models). Some of these mathematical models are chiefly concerned with forecasting the future of the epidemics, and are devoid of the effectiveness of health policy interventions in dampening the spread of the disease^3^; others (such as^4,5^) also incorporate possible health policy framework in the models. Even though these studies have provided some insightful approaches to the containment of the disease, one aspect that to the best of our knowledge appears to often been ignored is the incorporation in these studies individuals-collective behavioural responses (we hereby refer to as community compliance).

The approaches prescribed by these studies in most cases are viewed from the perspectives of public health authorities. Some of these models (see, for example^6–8^) look at the impact of social distancing and face mask use on the spread of the disease without incorporating explicitly community compliance with such measures, and therefore the associated impact on the disease transmission; for instance, the boycotts and demonstrations against such measures in some jurisdictions attest to the vital role individuals play in the disease spread. The introduction of the pharmaceutical measures does not offer complete guarantee for disease containment as individuals in the population have to decide whether to be vaccinated or not. Moreover, vaccinations do not provide perfect immunity in some cases. Factors as these contribute to the spread of the disease; for example^9–12^ showed that a well-intentioned public health policy may fail if individual behavioural responses to disease infectivity are not factored into policy framework. This suggests that public health policy should be contextualized within individuals’ reaction to disease outbreak. These are important aspects worth taking note of in addressing the transmission and control of COVID-19^13–16^. To this end, this study addresses the COVID-19 infectivity and control within optimization framework, where the focal parameter is community compliance rate combined with vaccination coverage and effective quarantine. Among others, we look at the efficiency and cost effectiveness of combining strategically these control measures. The remainder of the paper is organized as follows:

“Methods” gives a description of the model; the model assumptions and parameters are described, epidemiological suitability of the model is established and discussed. It also discusses the optimal control problem; the existence of an optimal control solution path and its characterization is proven. The numerical analysis and its discussion are presented in “Result and Discussion”. Here, we estimated the values of the model parameters that are not currently reported in the literature; this estimation is based on some reasonable assumptions. We then use these estimates to numerically simulate the optimality system. Also, the section discusses the various strategies proposed in the study; their efficiency and cost effectiveness calculated. Finally, "Conclusion" summarizes our findings, and policy recommendations provided thereof.

## Methods

### Mathematical model formulation

**Figure 1:**
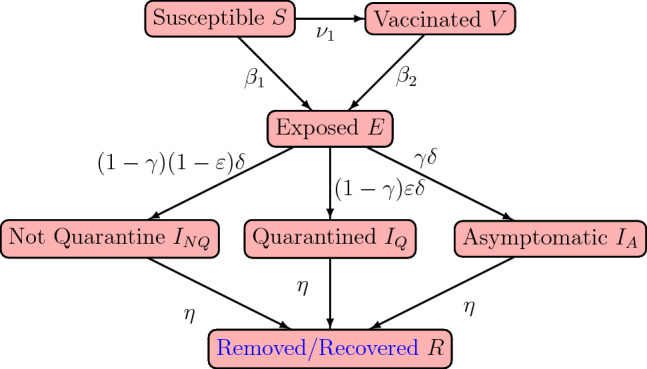
Flow diagram demonstrating the COVID-19 transmission model.

We employed a disease compartmental model in our study. Consider the flow diagram presented in Fig. 1: the figure is a schematic representation of the COVID-19 transmission mechanism within a population. The modelling framework assumes the absence of vital statistics such as birth and death rate; demographic parameters such as birth and natural death rates can be excluded from mathematical models when investigating disease dynamics occurring within few weeks or months (see the works by, for example^6,17–22^ . In particular, the works outlined by^6,18,20–22^ provided studies relating to COVID-19 by considering dynamic systems excluding demographic effects such as birth and natural death rate. Moreover, mathematical models without demographic parameters have been used extensively in studying dynamics of disease epidemics. Models of this nature (epidemic models) are used to model rapid outbreaks that happen in less than a year^19^. For this reason, given the time frame considered for the purpose of the study, (approximately 4 months) excluding these demographic parameters from our proposed model is a reasonable assumption. Moreover, we modelled human responses to the epidemic period of an infectious outbreak: as such, the time period of an outbreak is relatively short compared to demographic (i.e., death and birth) processes.

The population is stratified into 7 mutually exclusive sub-populations: Susceptible (*S*), Vaccinated (*V*), Exposed (*E*), Infected and Symptomatic but Not Quarantined ($I_{NQ}$), Infected and Symptomatic but Quarantined ($I_{Q}$), Infected and Asymptomatic ($I_{A}$), and Removed/Recovered (*R*). *S* captures the individuals in the population that have not yet been exposed to the disease and are susceptible; *V* is the group within the population that have received vaccination. $I_{NQ}$ are those infected and symptomatic individuals that are not quarantined. These individuals interact with the general population; they may or may not be aware of their status. $I_{Q}$ is the infected group of individuals that are aware of their status and are removed from the general public. The quarantine could be in a hospital, residence or any facility that serves that purpose. This group does not interact with the general population and it is a measure imposed by the health care authority. The $I_A$ group comprises of individuals who are infected but do not exhibit symptoms of the disease and are infectious, and the *R* group are those eliminated from the other disease categories by way of treatment or disease induced death. Recovered individual susceptibility period to disease (or to be proned reinfection) is very high-usually after 40 weeks^23,24^. Consequently, we assume that recovered individuals remain immune to the disease during the epidemic period, given the duration of time under discussion.

$\nu _1$ is the per capita rate of vaccination. We assumed that the time period of the disease dynamics does not account for waning period of vaccine where a proportion of the vaccinated population loses immunity and become susceptible. $\nu _1$ is time dependent, and is a control variable. Susceptible and vaccinated individuals enter the exposed group at the rate $\beta _1$ and $\beta _2$, respectively. $\beta _1$ and $\beta _2$ are modelled as

1 $$\begin{aligned} \beta _1=(1-x_t \kappa )\frac{\lambda _{I_{NQ}} I_{NQ}+\lambda _{I_A}I_A+(1-\nu _{2,t})\lambda _{I_Q}I_Q}{N-\nu _{2,t} I_Q} \end{aligned}$$

and

2 $$\begin{aligned} \beta _2=(1-\omega )\beta _1. \end{aligned}$$

Here, we assume that transmission is frequency dependent. The denominator accounts for that part of the population that does not contribute to infection, which is the quarantine population $I_Q$. $0\le \nu _2\le 1$ measures the effectiveness of quarantine. For instance, a value of $\nu _2=0$ implies ineffective quarantine measures in the prevention of transmission of the disease and $\nu _2=1$ suggests highly effective quarantine measures. It captures the effort by health authorities to make sure that quarantine measures produce expected results and reflects the level of resources committed; it is the cost associated with implementing quarantine measures. This parameter, therefore, evolves with time and it is a control variable within our modelling set up.

Vaccination induces protection against the disease and can reduce or eliminate the incidence of infection. Thus, we assume that $\beta _2\le \beta _1$, which is captured by $0\le \omega \le 1$. $\lambda _{I_{NQ}}, \lambda _{I_A}$, and $\lambda _Q$ are the effective contact rates associated with the $I_{NQ}, I_A$, and $I_Q$, respectively. $0\le \kappa \le 1$ measures the effectiveness of community compliance and $0\le x \le 1$ captures the community compliance rate for susceptible and vaccinated individuals. Compliance could be adhering to social distance, hand sanitizing, face mask use, and any other preventive protocols within the collective control by individuals. The product $x\kappa$ indicates the overall effect of the community compliance, where $\kappa$ serves as the lever for such effectiveness; high values of $\kappa$ implies high effectiveness of community compliance. As an example, suppose a representative community is complying with face mask usage, say 70% of the time in a time period; the effectiveness of this action is measured by $\kappa$ and the success $70\%$ times of usage per the period is captured by *x*. Within the modelling framework, *x* is one of the system’s control variables, hence it evolves with time.

We assume that $\gamma$ proportion of individuals remain asymptomatic after infection, and that on average the latency period is $\frac{1}{\delta }$—the number of days between the event of individuals transitioning from the exposed compartment to the infectious compartment ($I_{NQ}, I_Q$, and $I_A$) is modelled as an exponential distribution. This implies that $1-\gamma$ is the proportion of individuals who become symptomatic after the latency period. Let $\varepsilon$ be the proportion of the quarantined symptomatic infectious individuals, so that $(1-\varepsilon )$ is the unquarantined proportion of symptomatic infectious individuals. Then $(1-\gamma )(1-\varepsilon )\delta E$, $(1-\gamma )\varepsilon \delta E$ and $\gamma \delta E$ enters the symptomatic and not quarantined, symptomatic and quarantined, and asymptomatic infectious compartment, respectively. A total of $\delta E$ individuals leave the exposed compartment. Finally, we assume an infectious period of $\frac{1}{\eta }$ days for symptomatic infectious and unquarantined, symptomatic infectious and quarantined, and asymptomatic infectious individuals. System (3) is the mathematical representation of the model. Table 1 lists the model parameters.

3 $$\begin{aligned} \begin{aligned} \frac{dS}{dt}&=-(\beta _1 +\nu _1)S,\\\frac{d V}{dt}&=-\beta _2V +\nu _1 S, \\ \frac{dE}{dt}&=-\delta E+\beta _1 S+\beta _2 V,\\ \frac{dI_{NQ}}{dt}&=-\eta I_{NQ}+(1-\gamma )(1-\varepsilon )\delta E,\\ \frac{I_Q}{dt}&=-\eta I_Q+(1-\gamma )\varepsilon \delta E, \\ \frac{I_A}{dt}&=-\eta I_A+\gamma \delta E,\\ \frac{dR}{dt}&=\eta I_{NQ}+\eta I_Q+\eta I_A, \end{aligned} \end{aligned}$$

with initial condition

4 $$\begin{aligned} X(0)=(S(0),V(0),E(0),I_{nq}(0),I_q(0),I_a(0),,R(0))\in \mathbb {R}_+^7. \end{aligned}$$

**Table 1:** Description of model parameters.

Parameter	Description
$\gamma$	Proportion of individuals who remain asymptomatic after infection
$1-\gamma$	Proportion of individuals who become symptomatic infectious after infection
$\delta$	Latency rate
$\varepsilon$	Proportion of quarantined symptomatic infectious individuals
$1-\varepsilon$	Proportion of unquarantined symptomatic infectious individuals
$\eta$	Removal rate for symptomatic infectious not quarantined, symptomatic
	infectious and quarantined, and asymptomatic infectious individuals
$\beta _1$	Susceptible-exposed transmission rate
$\beta _2$	Vaccinated-exposed transmission rate
$\kappa$	Measure of effectiveness of compliance rate
$\omega$	Measure of effectiveness of vaccine
$\lambda _{I_NQ}$	Effective contact rate of symptomatic infectious not quarantined individual
$\lambda _{I_Q}$	Effective contact rate of symptomatic infectious quarantined individuals
$\lambda _{I_A}$	Effective contact rate of asymptomatic infectious individuals
Control parameters (or variables)	Description
$\nu _1$	Per capita rate of vaccination
$\nu _2$	Measure of effectiveness of quarantine controls
*x*	Community compliance rate

### Positivity and boundedness

To prove that System (3) satisfies epidemiological requirements, observe that the System satisfies the property

5 $$\begin{aligned} {\left\{ \begin{array}{ll} &{}\frac{dS}{dt} \ge -\beta _1S,\\ &{}\frac{dV}{dt} \ge -\beta _2 V,\\ &{}\frac{dE}{dt} \ge -\delta E,\\ &{}\frac{d I_{NQ}}{dt} \ge -\eta I_{NQ},\\ &{}\frac{dI_Q}{dt} \ge -\eta I_Q,\\ &{}\frac{dI_A}{dt} \ge -\eta I_A. \end{array}\right. } \end{aligned}$$

Now, consider the first expression in the inequality above:

$$\begin{aligned} \frac{dS}{dt}\ge -\beta _1S, \end{aligned}$$

then we have

$$\begin{aligned} S(t)\ge S(0)\exp \left( -\int _{0}^{t}\beta _1 ds\right) . \end{aligned}$$

Since $S(0)\ge 0$, *S*(*t*) is positive for all $t>0$. This property holds for the other expressions in the above inequality, hence $V(t), E(t), I_{NQ}(t),I_Q(t), I_A(t)$ are positive for all $t>0$. Also *R*(*t*) is positive for all $t>0$, since $R(t)=N(t)-S(t)- E(t) -I_{NQ}(t) -I_Q(t) - I_A(t)$, where *N*(*t*) is the total population at time *t* assumed as constant.

#### Theorem 1

*Given that the initial data* *X*(0) *of System*3*are positive, its solutions are positive for all* $t>0$.

We can show that the right hand side of the inequality has bounded solutions, therefore making use of Gronwall’s inequality^25,26^, we deduce the following:

#### Lemma 1.1

*The region* $\Omega =\{(S,V,E,I_{NQ},I_Q,I_A,R)\in R^7_+: N=N'\}$ *is positively invariant for System* (3) *and attracts all solutions*. $N'$ *is some positive constant*.

### Optimal strategy

Assessing the impact of the interplay of the collective efforts by individuals (examples, social distancing, face mask use, etc.) and health authority (examples, vaccination, quarantine, hospitalization, etc.) on the disease transmission and control requires that decision makers choose control parameters such that social and economic cost of the disease over the epidemic period is minimized. The control parameters within this modelling framework are $\{\nu _i\}_{ i=1,2}$ and *x*. The parameter $\nu _i$ are within the control of health authority. As noted in the preceding section, $\nu _1$ is the per capita rate of vaccination and $\nu _2$ is the effectiveness of instituted quarantine measures. They capture the associated cost of these control measures, for example, cost of materials, facilities, and labor used. *x* is the collective contributions of the individuals toward the reduction of the spread of the disease. We define the objective functional as

6 $$\begin{aligned} J(x_t,\nu _{1,t},\nu _{2,t})&=\int _{0}^{T}e^{-\rho t}f\left( x_t,\nu _{1,t},\nu _{2,t}\right) dt, \end{aligned}$$

where $0\le t\le T$. The function $f(\cdot )$ is the associated social and economic cost that have to be minimized over the epidemic period, and is governed by

7 $$\begin{aligned} f(x, \nu _1,\nu _2)=A_1 I_{NQ,t}+A_2 I_{Q,t}+A_3 I_{A,t}+B x^2_t+\sum _{i=i}^2 C_i \nu ^2_{i,t}, \end{aligned}$$

and the control set

$$\begin{aligned} Z=\{(x,\nu _1,\nu _2)|\text { each controll is Lebesgue measurable on } [0 \quad T],0\le x,\nu _1,\nu _2\le 1\}. \end{aligned}$$

$\{A_i\}_{i=1,2,3}$ are the fixed cost associated with being infected by the disease, which includes the pain, discomfort and lost labor income or leisure; *B* is the fixed cost corresponding to community compliance with health directives, and $\{C_i\}_{i=1,2}$ are the fixed cost for vaccination and effectiveness of quarantine. Observe that the cost describing the controls are expressed as quadratic function of the controls ($Bx^2_t, C_1\nu _{1,t}^2,C_2 \nu _{2,t}^2$). We specify the cost function by choosing a linear function for the infection cost and quadratic function for controls cost in line with work done in^27–31^. The choice has the implication that highlights the increasing cost associated with each of the controls. Implying the marginal cost associated with each of the efforts to reducing infection is increasing in nature. This formulation of the cost function assumes that the cost per treatment of COVID-19 per infected per unit time is an increasing cost; the vaccination cost is an increasing function of the coverage of the vaccination program; the compliance rate and effort put into the effectiveness of the quarantine measure is also an increasing cost. Thus, the associated marginal cost to the controls are increasing as increase in the control measures adds to the cost of their execution.

The parameter $0\le \rho \le 1$ is the discount factor. The discount factor accounts for the present value of the future cost of the intervention by the individuals and health authority. Our modelling framework assumes a constant non-zero discount factor.

#### Existence of optimal control

To demonstrate the existence of optimal control for the control system we use the theorem outlined in^32^, Theorem III.4.1) which requires we show that the following properties hold:

1. The control set *Z* and associated state variables is non empty.
2. The control set *Z* is convex and closed.
3. The state system is bounded by a linear function in the state and control on the right hand side.
4. The integrand of the objective functional is concave on *Z*.
5. There exist constants $c_1>0,c_2>0,$ and $\theta >1$ such that the integrand of the objective functional satisfies
  $$\begin{aligned} A_1 I_{NQ}+A_2 I_Q+A_3 I_A+B x^2+\sum _{i=i}^2 C_i \nu ^2_i\ge c_1\left( |x|^2+\sum _{i=i}^2|\nu _i|^2\right) ^{\frac{\theta }{2}}-c_2. \end{aligned}$$

Using the result in^33^, we can demonstrate that system (3) has solution with bounded coefficients and by definition the control set *Z* is convex and bounded, thus conditions 1 and 2 are met. From Theorem 1 and Lemma 1.1 the state System (3) has a bounded solution on the finite time interval $[0 \quad T]$; for this reason the right hand side of the state system (3) satisfies condition 3. Finally, the integral of the objective functional is convex on the control set, with an additional property that the integrand of the objective functional is bounded below by

$$\begin{aligned} c_1\left( |x|^2+\sum _{i=i}^2|\nu _i|^2\right) ^{\frac{\theta }{2}}-c_2 \end{aligned}$$

for $c_1>0,c_2>0$ and $\theta >1$, since the state variables are bounded. Furthermore, $\rho \ge 0$. Against this background, We formally present the following theorem:

##### Theorem 2

*Consider the control problem with system* (3). *There exist* $\zeta ^*=(x^*,\nu _1^*,\nu _2^*)\in Z$ *such that*

$$\begin{aligned} \underset{(x,\nu _1,\nu _2)\in Z}{ min}\ J(x,\nu _1,\nu _2)=J(x^*,\nu _1^*,\nu _2^*). \end{aligned}$$

#### Characterizing the optimal control

##### Theorem 3

*Given optimal control* $\zeta ^*= (x^*,\nu _1^*,\nu _2^*)$ *and solutions* $(S^*,V^*,E^*,I_{NQ}^*,I_{Q}^*,I_A^*)$ *of the corresponding state system* (3), *there exist adjoint (or costate) variables* $\lambda _1,\lambda _2,\lambda _3,\lambda _4, \lambda _5,\lambda _6$ *and* $\lambda _7,$ *satisfying*

8 $$\begin{aligned} \begin{aligned} \frac{d\lambda _1}{dt}&=\rho \lambda _1+(\beta _1+\nu _1)\lambda _1-\nu _1\lambda _2-\beta _2\lambda _3,\\ \frac{d\lambda _2}{dt}&=\rho \lambda _2-\psi \nu _1\lambda _1+(\beta +\psi \nu _1)\lambda _2-\beta _2\lambda _3,\\ \frac{d\lambda _3}{dt}&=\rho \lambda _3+ \delta \lambda _3-(1-\gamma )(1-\varepsilon )\delta \lambda _4-(1-\gamma )\varepsilon \delta \lambda _5-\gamma \delta \lambda _6,\\ \frac{d \lambda _4}{dt}&=\rho \lambda _4-A_1+\frac{\partial \beta _1}{\partial I_{NQ}} (S \lambda _1+(1-\omega )V\lambda _2)-\frac{\partial \beta _1}{\partial I_{NQ}} (S+(1-\omega )V)\lambda _3+\eta _1\lambda _4-\eta _1\lambda _7,\\ \frac{d \lambda _5}{d t}&=\rho \lambda _5-A_2+\frac{\partial \beta _1}{\partial I_Q}(S\lambda _1+(1-\omega )V\lambda _2) -\frac{\partial \beta _1}{\partial I_Q}(S+(1-\omega )V)\lambda _3 + \eta _5\lambda _7-\eta _2\lambda _7,\\ \frac{d \lambda _6}{dt}&=\rho \lambda _6-A_3+\frac{\partial \beta _1}{\partial I_A}( S\lambda _1+(1-\omega )V\lambda _2)-\frac{\partial \beta _1}{\partial I_A}(S+(1-\omega )V)\lambda _3+\eta _3\lambda _6-\eta _3\lambda _7,\\ \frac{d\lambda _7}{dt }&=\rho \lambda _7, \end{aligned} \end{aligned}$$

*where*

9 $$\begin{aligned} {\left\{ \begin{array}{ll} \frac{\partial \beta _1}{\partial I_{NQ}}&{}=(1-x \kappa )\frac{\lambda _{I_{NQ}}}{N-\nu _2 I_Q}\\ \frac{\partial \beta _1}{\partial I_A}&{}=(1-x\kappa )\frac{\lambda _{I_A}}{N-\nu _2 I_Q}\\ \frac{\partial \beta _1}{\partial I_Q} &{} = (1-x\kappa )\left[ \frac{(1-\nu _2)\lambda _{I_{Q}}}{N-\nu _2 I_Q}+\frac{(\lambda _{I_{NQ}}I_{NQ}+\lambda _{I_{A}}I_{A}+(1-\nu _2)\lambda _{I_{Q}}I_{Q})\nu _2}{(N-\nu _2 I_Q)^2}\right] \end{array}\right. }, \end{aligned}$$

*with transversality conditions*

10 $$\begin{aligned} \lambda _{i}(T)=0, \text { for } i=1,2,\ldots ,7. \end{aligned}$$

In addition, the optimal controls satisfy

11 $$\begin{aligned} \begin{aligned}{}&2 B x^* -g_x(\xi ^*) (S^* \lambda _1 -(1-\omega )V^* \lambda _2)+g_x(\xi ^*)( S^*+ (1-\omega )V^*)\lambda _3=0,\\&2 C_1\nu _1^*-(S^*-\psi V^*)\lambda _1-(\psi V^*-S^*)\lambda _2=0,\\&2C_2\nu _2^*-g_{\nu _2}(\xi ^*)(S^*\lambda _1-(1-\omega ))V^*\lambda _2)+g_{\nu _2}(\xi ^*)(S^*+(1-\omega )V)\lambda _3=0, \end{aligned} \end{aligned}$$

*where*

12 $$\begin{aligned} {\left\{ \begin{array}{ll} g_x(\xi ^*)={\frac{\partial \beta _1}{\partial x}}\big |_{\xi =\xi ^*}=-\kappa \left( \frac{\lambda _{I_{NQ}}I_{NQ}^*+\lambda _{I_A}I_A^*+(1-\nu _2)\lambda _{I_Q}I_Q^*}{N-\nu _2^* I_Q^*}\right) \\ g_{\nu _2}(\xi ^*)={\frac{\partial \beta _1}{\partial \nu _2}}\big |_{\xi =\xi ^*}=-(1-x^*\kappa )\left( \frac{\lambda _{I_Q}I_Q^*}{N-\nu _2I_Q^*}-\frac{(\lambda _{I_{NQ}}I_{NQ}^*+\lambda _{I_A}I_A^*+(1-\nu _2^*)\lambda _{I_Q}I_Q^*)I_Q^*}{N-\nu _2^*I_Q^*}\right) \end{array}\right. }, \end{aligned}$$

*and* $$\xi ^*=(x^*,\nu _1^*,\nu _2^*,I_{NQ}^*,I_A^*,I_Q^*)$$.

##### *Proof*

We prove Theorem 3 by first recognizing that the necessary conditions an optimal control must meet is derived from the Pontryagin’s Maximum Principle^34^. We set the current-value Hamiltonian (see, for example^35^ for a quick review) for the control problem as follows:

13 $$\begin{aligned} H=f(x,\nu _1,\nu _2)+\lambda _1 {\dot{S}}+\lambda _2{\dot{V}}+\lambda _3 {\dot{E}}+\lambda _4{\dot{I}}_{NQ}+\lambda _5 {\dot{I}}_Q+\lambda _6{\dot{I}}_{A}+\lambda _7 {\dot{R}}, \end{aligned}$$

with optimality condition

14 $$\begin{aligned} \frac{\partial H}{\partial x}=\frac{\partial H}{\partial \nu _1}=\frac{\partial H}{\partial \nu _2}=0. \end{aligned}$$

We note that ${\dot{S}}=\frac{\partial H}{\partial S},{\dot{V}}=\frac{\partial H}{\partial V}, {\dot{E}}=\frac{\partial H}{\partial E},{\dot{I}}_{NQ}=\frac{\partial H}{\partial I_{NQ}},{\dot{I}}_Q=\frac{\partial H}{\partial I_Q},{\dot{I}}_A=\frac{\partial H}{\partial I_A }$, and ${\dot{R}}=\frac{\partial H}{\partial R }$. The conditions defining the adjoint variables $\lambda _{i}$,$i=1,2,\ldots ,7$ are given as

15 $$\begin{aligned} {\left\{ \begin{array}{ll} \frac{d\lambda _1}{dt}=\rho \lambda _1 -\frac{\partial H}{\partial S}\\ \frac{d\lambda _2}{dt}=\rho \lambda _2 -\frac{\partial H}{\partial V}\\ \frac{d\lambda _3}{dt}=\rho \lambda _3 -\frac{\partial H}{\partial E}\\ \frac{d\lambda _4}{dt}=\rho \lambda _4 -\frac{\partial H}{\partial I_{NQ}}\\ \frac{d\lambda _5}{dt}=\rho \lambda _5 -\frac{\partial H}{\partial I_Q}\\ \frac{d\lambda _6}{dt}=\rho \lambda _6 -\frac{\partial H}{\partial I_A }\\ \frac{d\lambda _7}{dt}=\rho \lambda _7 -\frac{\partial H}{\partial R } \end{array}\right. }. \end{aligned}$$

$$\square$$

##### Remark 1

To characterize the optimal control we need to solve Eq. (11) on the interior of the control set and follow the approach in^27,36–38^. The bounds on the controls are then imposed on the solution. Observe from Eq. (11) that obtaining explicit expressions for the controls *x* and $\nu _2$ is unobtainable and that has to be computed numerically. The control $\nu _1$ has its expression as

16 $$\begin{aligned} \nu _1=\max \left\{ \min \left\{ 1,\frac{\lambda _1(S^*-\psi V^*)+\lambda _2(\psi V^*-S^*)}{2 C_1}\right\} ,0\right\} . \end{aligned}$$

##### Remark 2

The costate variables $\{\lambda _i\}_{i=1,2,\ldots ,7}$ are current value marginal cost associated with each of the disease states. These costs are obtained by solving System (8). The resulting solution evaluated at the optimum is the optimal marginal cost $\{\lambda _i^*\}_{i=1,2,\ldots ,7}$ of the disease states.

### Ethical approval and consent

 All authors have been personally and actively involved in substantial work leading to the paper, and will take public responsibility for its content. The method employed in this work are in accordance with all the relevant guidelines and regulations. Observe that the method outlined above does not involve experimentation with humans.

## Result and discussion

Our goal in this section is to provide the numerical simulation of the optimality system—System (3), (8), and (11). We use the steepest descent approach prescribed in^39^ in simulating the optimality system; as also used in^27,30,40,41^. Adopting the MATLAB code in^42^, we iteratively solve the optimality system using the Runge-Kutta fourth order procedure. Starting with a guess for the controls, the state system (3) is solved forward in time and then the co-state system (8) backward in time due to the transversality conditions.

Since the literature does not contain all the values of the baseline parameters in the state system (3), some of the parameters in the model are estimated. The estimation is done in two folds:

1. The case where vaccination and quarantine measures are in place. This is to allow for studies on how collective individual behaviors affect the dynamic of the disease. This is discussed in “Community influence on disease dynamics via compliance rate”. Here, the controls, compliance rate (*x*), vaccination ($\nu _1$) and effective quarantine ($\nu _2$) parameters are estimated. The objective is to show why although infections happens at the population level, decisions to prevent infections lie at the individual level. It is therefore imperative these additional dynamics are incorporated into disease models. Note that the compliance rate within this modelling framework is at the community level. We focus on the collective effort on compliance of individuals in the population (community).
2. The case where vaccination, quarantine, and compliance measures are not present in the population. This permits combination of the different controls, which then allows us to study their impacts on the disease dynamics and the associated social and economic costs, as the objective of the study dictates. None of the controls are estimated. “Control strategies” has detailed discussion on this.

The unestimated baseline parameters are presented in Table 2. We assumed that the disease has short epidemic period and that when one is vaccinated, the vaccine confers permanent immunity. Since the data set used for our study is based on COVID-19 cases in Nigeria, we assume the COVAX vaccine (the vaccine used in Nigeria) efficacy $\omega$ as 91% (which is same as that of BNT162b2—91% (89.0–93.2%))^43^) as at the time of study there is not conclusive evidence on the vaccine efficacy; this assumes vaccinated individuals have received two doses. The fixed costs $A_1,A_2,A_3,B,$ and $C_2$ are arbitrarily chosen. The value for $\rho$ is converted from the recommended annual rate of 3.5%^44^ to its daily equivalent. We assume the same removal rate $\eta =1/9$ for each of the infectious classes. The implication of removal/recovered rate is that the transitioning from the infected compartments to the Removed/Recovered compartment occurs either via the disease induced death or recovery from the disease upon infection. So, setting $\eta =1/9$, implies that infected individuals recover or die on average 9 days upon infection. Given no conclusive report on the length of days to die from the disease, coupled with no data on the removal rate (per definition in this study), and the objective of our studies, we deemed it reasonable to assume the above value for $\eta$. This is consistent with a published estimate of 9.78 ($95\%$ confidence interval 8.45–21.78) days after the onset of symptoms^45^. Furthermore, several studies have found a quicker time to recovery in African countries, probably reflecting the younger affected population (see^46,47^). Now, uncertainty quantification in mathematical models is crucial in assessing their accuracy as the obtained measurements upon which their calibrated are in most cases noisy. The goal of statistical analysis of these models is to measure the uncertainty in them, which occurs at the model estimation stage via model parameter estimation. In this section, we quantify such uncertainty in System (3) using Markov Chain Monte Carlo (MCMC). The estimation is based on the daily reported COVID-19 cases on Nigeria^48^. We employed the MCMC estimation scheme based on the Delay Rejection Adaptive Metropolis^49^ by adopting the MATLAB package mcmcrun presented by^50^. The likelihood function of the observed state, number of daily infections, is assumed as normal distribution. The prior distributions of the parameters are assumed normally distributed. The MCMC is run by first obtaining the Least Squares Estimates of parameters and the corresponding covariance matrix. The estimates are then used as initial guess in the MCMC algorithm, and the covaraince matrix used as the initial proposal covariance for the MCMC samples. Three runs of chains were run. The first run was used as the initial chain for the second, and the second run was used for the initial chain of the third. In all, there were 75,000 simulations with each run of chains constituting 25,000 simulations.

We assessed the goodness of fit by employing the normalized mean square error (NMSE), expressed as follows:

$$\begin{aligned} NMSE=1-\frac{|| (\text {actual number of cases})-(\text {predicted number of cases})||^2}{||(\text {actual number of case}s)-(\text {mean of actual number of cases})||^2}, \end{aligned}$$

where $||\cdot ||$ denotes the 2-norm of a vector. NMSE is in the interval $[-\infty , 1]$, where $-\infty$ indicates a bad fit and 1 a perfect fit.

**Table 2:** Unestimated baseline parameters.

Parameter	Value	References
$$\delta$$	$$\frac{1}{5.2}$$ daily	^51^
$$\eta$$	$$\frac{1}{9}$$ daily	^45–47^
$$\omega$$	0.91	Assumed
*N*	211184869	^52^
$$\rho$$	0.009% daily	^44^
$$C_1$$	$3.7	^53^

### Community influence on disease dynamics via compliance rate

Health policies prescribed based on traditional mathematical disease models are from public health authorities perspectives devoid of how individuals respond to such prescriptions; they are concerned with forecasting future epidemic, parameter estimations for strategic choices, and the mechanism for their disease spread. The economic epidemiology models is a combination of traditional mathematical epidemiology and economic choice; they incorporate the interaction of economic incentives and behavioral responses to address how diseases are transmitted, and how individuals’ incentives affect disease spread and cost of health interventions.

This section compares numerical results under traditional (classical) mathematical and economic epidemiology modelling framework; the goal is to show how the results and conclusions under these respective modelling framework differ. We achieve this by setting the compliance rate *x* as the control variable in the optimal control problem—this captures the individuals’ collective choice via community compliance. This is to say the community compliance rate in our modelling setting is within the remit of individuals, and this rate has to be determined optimally subjected to economic incentives. Against this background, the numerical results under traditional mathematical framework (system 3) are obtained by fixing—not evolving with time—all model parameters. The economic epidemiology framework, assumes fixed parameters for all parameters, except compliance rate *x*, which is a control variable. Summarizing, all model parameters are fixed, including community compliance rate *x*, in the traditional mathematical model (system 3). However, in the economic epidemiological model—the optimal control problem (System (3),(8), and (11)—we fixed all parameters except community compliance rate *x*. This allows for incorporation of collective decision making on the part of individuals.

We make use of the daily reported COVID-19 cases on Nigeria^48^ from March 4, 2021 to June 27, 2021 in the parameter estimation procedure for this section. By considering a time window of March 4, 2021 to June 27, 2021, we assume that vaccination program is in effect and concurrent with various non-pharmaceutical interventions; which in our study are the compliance rate and quarantine effectiveness. Table 3 presents the estimates of the model parameters in the state system 3 with their corresponding estimation errors. The initial state values used are $I_{NQ}(0)=200, I_Q(0)=71, I_A(0)=100$, where that of *E*(0) and *R*(0) are, respectively, estimated as 121.3 and 184.95, with corresponding estimate for $S(0)=211183465$. Note that in the numerical simulations the values of *E*(0) and *R*(0) are approximated as 121 and 185, respectively.

**Figure 2:**
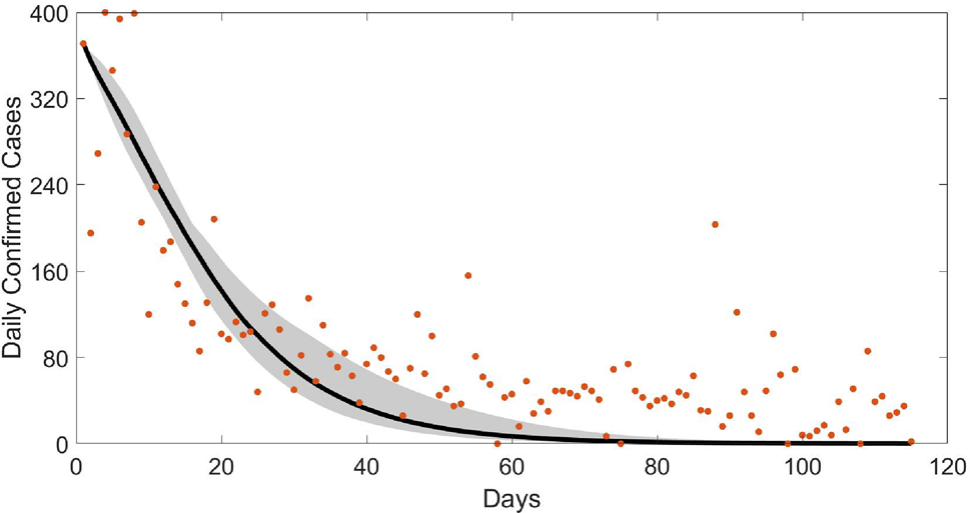
Fitted (black line) new daily confirmed COVID-19 cases using Nigeria time series data (red dots) from March 4, 2021 to June 27, 2021. We assume the presence of all controls ($x,\nu _1, \nu _2)$. The grey area is the 95% confidence bands. Initial state values: $S(0)=211183465, I_{NQ}(0)=200, I_Q(0)=71, I_A(0)=100, E(0)=121, R(0)=185$.

**Figure 3:**
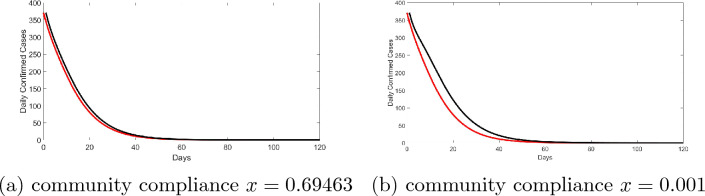
A comparison of community influence via compliance rate: with optimal control path (red curve) and without optimal control (black curve). The compliance rate (*x*) is the control variable. The values of the other model parameters are given in the tables above. Initial state values: $S(0)=211183465, I_{NQ}(0)=200, I_Q(0)=71, I_A(0)=100, E(0)=121, R(0)=185$.

Figure 2 shows the fitted model. Now, using these estimated values, we then simulated the optimality system using the compliance rate as the control. Figure 3 compares the daily infections obtained from traditional mathematical disease model (black line) where community compliance was not explicitly incorporated and the economic epidemiological model (red line) where economic choice in the form of community compliance is explicitly incorporated via optimal control framework. Figure 3a compares the simulation result based on traditional mathematical model using the estimated value of compliance rate ($x=0.69463$) and that of the economic epidemiological model. Observe the difference in the results; we record high values of infections from the traditional mathematical model. As an experiment, when one reduces the value of *x*—see Fig. 3b where $x=0.001$—the gap increases. This is reflective of the cost imposed on individuals in complying with non-pharmaceutical directives. Over the epidemic period, individuals make collective decisions to minimize this cost. This result mirrors the findings in^9,10,54^. Implying, health policy decisions based on estimates from traditional mathematical model may not yield required results. This highlights that in the effort of curbing disease epidemic, contextualizing health policies is imperative. And that COVID-19 control is very much dependent on the collective behavior of the population.

**Table 3:** Estimated parameters, where no presence of controls are considered: MCMC statistics, 25,000 number of simulations.

Parameters	Mean	Standard deviation	Markov chains error
$\lambda _{I_{NQ}}$	0.12849 daily	0.11878	0.016925
$\lambda _{I_Q}$	0.14773 daily	0.11812	0.018374
$\lambda _{I_A}$	0.51645 daily	0.37632	0.059863
$\gamma$	0.46963	0.2225	0.036122
$\varepsilon$	0.094872	0.10845	0.022947
$\nu _2$	0.44511	0.29083	0.056373
*x*	0.69463	0.2445	0.039404
$\nu _1$	0.20956 daily	0.15751	0.031465
$\kappa$	0.64311	0.24491	0.034543
$E_0$	121.31	17.54	2.1927
$R_0$	184.95	51.061	7.4022

### Control strategies

As noted in the introductory section of this document, the purpose of this study is to investigate some proposed strategies—Table 4—for combating the spread of COVID-19. We are also interested in the cost effectiveness of these decisions. For this purpose the estimation procedure considers a time series window from March 16, 2020 to June 29, 2020. This allows us to address the above objective—this is approximately the time when COVID-19 is officially recorded, and vaccination program and various non-pharmaceutical interventions were not in effect, which in our study are the compliance rate and quarantine effectiveness.

Obtaining parameter estimates based on the above allows us to determine baseline values for the fixed parameters. For the purpose of our study the parameters that vary in the model are $x, \nu _1,$ and $\nu _2$; these are the control variables.

**Table 4:** Different control strategies.

Strategy	Description
Strategy 1	Compliance rate combined with vaccine and effectiveness of quarantine
Strategy 2	Vaccine combined with effectiveness of quarantine
Strategy 3	Compliance rate combined with vaccination
Strategy 4	Compliance rate combined with effectiveness of quarantine

**Figure 4:**
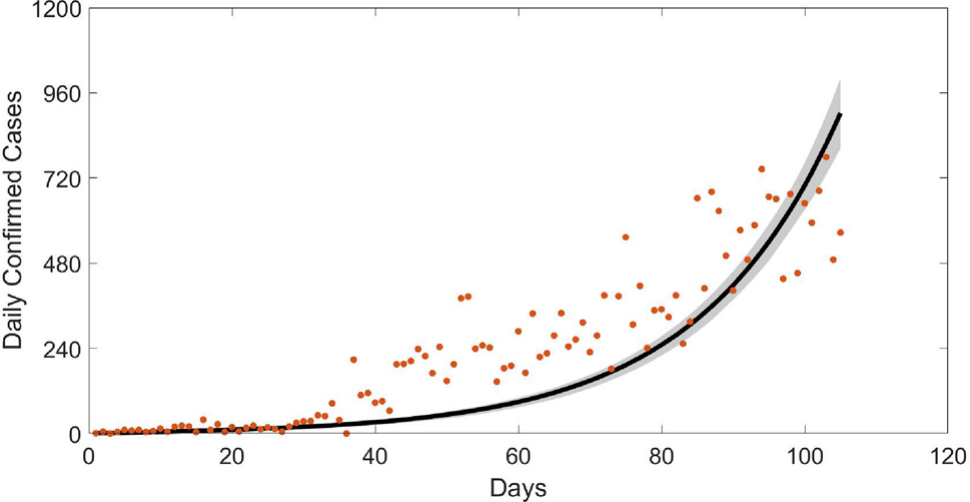
Fitted (black line) new daily confirmed COVID-19 cases using Nigeria time series data (green dots) from March 15, 2020 to June 29, 2020. We assume the absence of all controls ($x,\nu _1, \nu _2)$. The grey areas are the 95% confidence bands.

The parameter values in Tables 2 and 5 are used in our analysis. Table 5 presents the means, standard deviations, and the MC errors of the parameters of interest; which are $\lambda _{I_{NQ}},\lambda _{I_Q},\lambda _{I_A},\gamma ,\varepsilon$ and $\kappa$. Figure 4 shows the fitted model-based on data set from March 16, 2020 to June 29, 2020. The model fitting (black line) was done using the respective means of the MC samples of the parameters. A 95% confidence band was constructed (grey area) to indicate the uncertainty in the model fit. The red dots are the real data on the daily confirmed cases in Nigeria. The initial state values are set at $S=211184868,I_{NQ}=1$ and the rest, $E(0)=V(0)=I_Q(0)=IA(0)=R(0)=0$.

**Figure 5:**
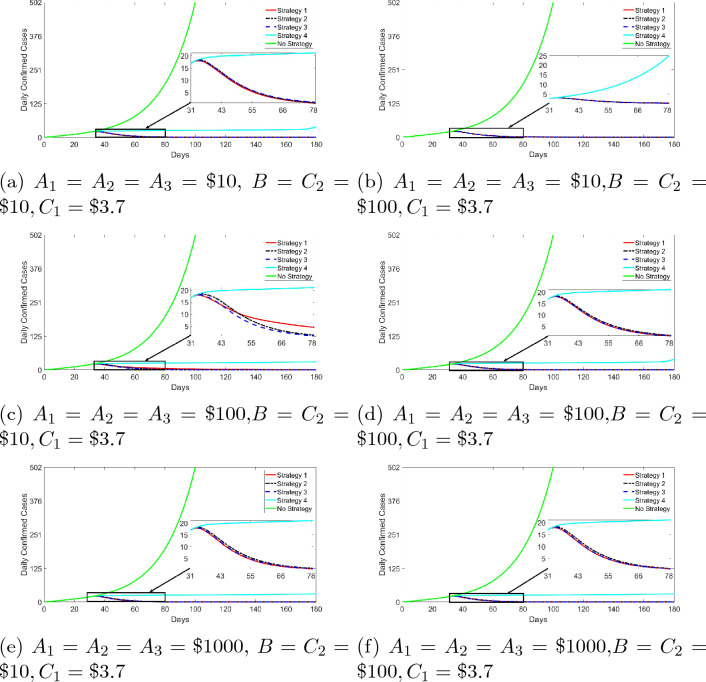
Daily confirmed infections for the different strategies. Parameter values used for graphs are presented in the tables above. $S(0)=211184868,I_{NQ}(0)=1,V(0)=E(0)=I_Q(0)=I_A(0)=R(0)=0$. We introduce strategies on the 31st day after the first record of the cases. The reference period is March 16, 2020 to June 19, 2020. Note that the green line is the path of COVID-19 cases without strategies and that line runs to 180 days—the graph presented a truncated version (cut-off is 100th day). This is to allow for showcasing the impact of the different control strategies (solid and dotted lines red, black, blue and cyan).

**Figure 6:**
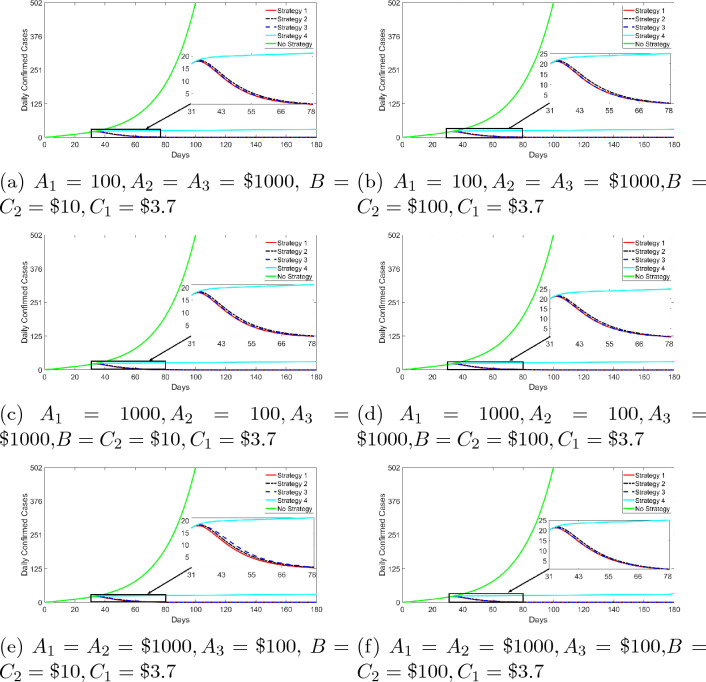
Daily confirmed infections for the different strategies. Parameter values used for graphs are presented in the tables above. $S(0)=211184868,I_{NQ}(0)=1,V(0)=E(0)=I_Q(0)=I_A(0)=R(0)=0$. We introduce strategies on the 31st day after the first record of the cases. The reference period is March 16, 2020 to June 19, 2020. Note that the green line is the path of COVID-19 cases without strategies and that line runs to 180 days—the graph presented a truncated version (cut-off is 100th day). This is to allow for showcasing the impact of the different control strategies (solid and dotted lines red, black, blue and cyan).

Having these estimates, we solve the optimality system by using the initial values of the state variables: $S=211184868,I_{NQ}=1, E(0)=V(0)=I_Q(0)=IA(0)=R(0)=0$. We run the simulation without control strategies from a period of 1 to 30 and then introduce the control strategies and run the simulation to the 180th day. We considered several values of the fixed cost in the objective functional (Eq. 6) to ascertain the sensitivity of the effectiveness of control strategies to these fixed costs; Figs. 5 and 6 demonstrate these sensitivity analyses. The sensitivity analyses consider two cases:

The first case assumes equal social and economic cost or burden of infections ($$A_1=A_2=A_3$$) and varying cost ($$B, C_1$$, and $$C_2$$) of the the controls; this is demonstrated in Fig. 5. The figure shows that when the fixed cost of infections are set equal to each other, couple with increasing values of the cost of the control, strategy 1 performs better than the other other strategies—except for cases where we use small values for cost of control ($$B=C_2=\$10, C_1=\$3.7$$) and relative high values of the fixed cost of infections ($$A_1=A_2=A_3=\$100$$), Fig. 5c. This means that there are particular combinations of the cost of controls and infection cost that results in other control strategies performing better than the strategy 1. However, it is fair to argue that since cost of non-pharmaceutical interventions (quarantine and community compliance in our case) could be way above $10 per person, we can therefore limit our study to values of *B* and $$C_2$$ to $100 and above. Following same chains of reasoning for the fixed cost associated with infections, we limit the findings to cost of $100 and above. By this, we conclude that strategy 1 (red curve) dominates, followed by strategy 3 (blue curve), and then strategy 2 (black curve) in reducing the spread of the disease.

The second case assumes unequal social and economic costs or burden of infections ($$A_1,A_2,$$ and $$A_3$$ are not necessarily equal) and varying costs ($$B, C_1$$, and $$C_2$$) of the controls; Fig. 6 compares the impact of the four control strategies using this case. We observe that strategy 1 (red curve) dominates in the reduction of the spread of the disease, followed by strategy 3 (blue curve), and then strategy 2 for the selected combined values of the various costs except for the case where the disease social and economic cost or burden associated with asymptomatic infection is less than that of symptomatic and unquarantine and quarantine infections accompany with small values of cost of controls, $$B=C_2=\$10$$ (Fig. 6e: we have strategy 2 dominating strategy 3). As pointed out in the first case (Fig. 6) that since the costs associated with non-pharmaceutical intervention are likely to be $100 and above, we conclude that by setting these costs along this range yields results consistent with what we observed in the first case: strategy 1 (red curve) dominates, followed by strategy 3 (blue curve), and then strategy 2 (black curve) in reducing the spread of the disease.

Summarizing, the sensitivity analysis indicates that for reasonable values of the cost of the controls $\nu _1, \nu _2$ and *x* control strategy 1 (red curve) is highly effective in eliminating COVID-19 from the population. This means that health policy directed at strengthening community compliance (this could be done via aggressive media campaigns) coupled with instituting effective quarantine and vaccination programs reduce the disease burden effectively. Even though strategy 4 (cyan curve) is the least effective control strategy it can reduce and probably eliminate the disease in the long run, thus making it desirable under certain settings where vaccination is hard to secure as in the case of some developing countries, of which Nigeria is not an exception; it reduces the disease burden in the long run. Thus, just effective compliance to non-pharmaceutical interventions may be sufficient. Strategy 3 (black curve) is the third best in curbing the spread of the disease. This strategy is most useful when health authority is unable to effectively put quarantine measures in place.

**Figure 7:**
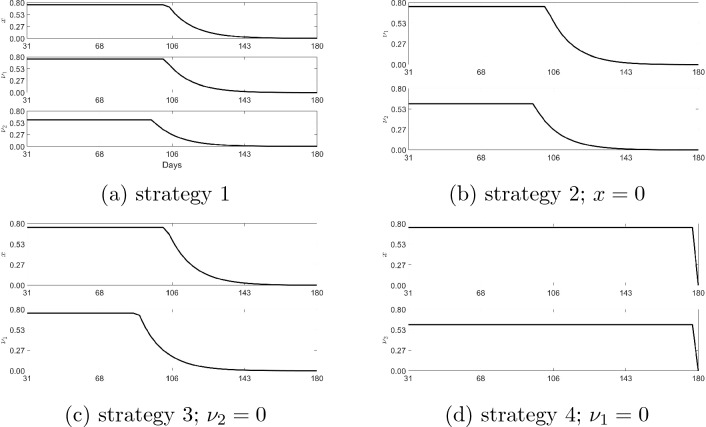
Control functions: we introduce strategies on the 31st day after the first record of the cases. The reference period is March 16, 2020 to June 19, 2020; the values of the other model parameters are given in the tables above. $A_1=\$100, A_2=A_3=\$1000,B=C_2=\$100,C_1=\$3.7$, $S(0)=211184868,I_{NQ}(0)=1,V(0)=E(0)=I_Q(0)=I_A(0)=R(0)=0$.

The ensuing subsections present efficiency analysis and incremental cost effectiveness ratio (ICER) for the proposed strategies. For this purpose, we assume $A_1=\$100, A_2=A_3=\$1000,C_1=\$3.7, B=C_2=\$100$, which corresponds to Fig. 6b. The corresponding optimal path for the control variables for each of the strategies are displayed in Fig. 7. The optimal paths are the optimal decisions corresponding to each time period. For example, consider the decision path for compliance rate (Fig. 7a): in period 0, it is optimal for community compliance rate to be 0.8; as time passes we see the rate decreasing with decreasing disease cases.

**Table 5:** Estimated parameters with presence of controls ($x=\nu _1=\nu _2=0$).

Parameters	Mean	Standard deviation	Markov chain error
$\lambda _{I_{NQ}}$	1.8781 daily	0.15644	0.023264
$\lambda _{I_Q}$	0.14338 daily	0.095303	0.013539
$\lambda _{I_A}$	0.091648 daily	0.041714	0.0080053
$\gamma$	0.67797	0.12564	0.022458
$\varepsilon$	0.82454	0.098291	0.018048
$\kappa$	0.45556	0.28041	0.038937

Markov Chains Monte Carlo statistics; 25,000 simulations.

### Efficiency analysis

This section compares the efficiency of the proposed strategies. We do this by introducing an efficiency index $\mathbb {I}$ that is a function of the cumulative number of daily confirmed cases during the time interval of the control strategies. This index is captured as

17 $$\begin{aligned} \mathbb {I}=\left( 1-\frac{\mathbb {E}_C}{\mathbb {E}_0}\right) \times 100, \end{aligned}$$

where the pair $(\mathbb {E}_C,\mathbb {E}_0)$ are the respective cumulative number of the new infectious individuals with and without the control strategies. The formulas^27,55,56^

18 $$\begin{aligned} {\left\{ \begin{array}{ll} \mathbb {E}_c=\int _t^T \delta E_cdt,\\ \mathbb {E}_c=\int _t^T \delta E_0dt,\\ \end{array}\right. } \end{aligned}$$

are the areas between the curve of the new infectious individuals and the time axis $[t \quad T]$. The pair $(E_c,E_0)$ are the exposed population size corresponding to infections with and without control strategies. The strategy with the biggest index is the best. Table 6 lists the efficiency index corresponding to each of the strategies $\mathbb {I}$. We see that even though $\mathbb {I}$ is highest for strategy 1, the gap is marginal.

**Table 6:** Efficiency Index of new cases across the different strategies: we introduce strategies on the 31st day after the first record of the cases.

Strategy	Total new cases over period	Efficiency index (%)
Strategy 1	28.9220	99.963
Strategy 2	33.2155	99.957
Strategy 3	29.1815	99.962
Strategy 4	508.6952	99.354
No control	78822	0.000

The reference period is March 16, 2020 to June 19, 2020; the values of the other model parameters are given in the Tables above. $S(0)=211{,}184{,}868,I_{NQ}(0)=1,V(0)=E(0)=I_Q(0)=I_A(0)=R(0)=0$.$A_1=\$100, A_2=A_3=\$1000,B=C_2=\$100,C_1=\$3.7$. Note that total new cases are obtained by summing new cases from day 31 to 180.

### Cost effectiveness analysis

When there are competing health strategies, it is best practice to choose that intervention with the minimum cost and the best outcome. Cost effectiveness analysis helps do exactly that. Efficiency analysis provides insight on the most efficient strategy regardless of its associated cost. It is crucial to know which strategy is the most efficient and its associated cost. This section ranks the strategies listed in Table 4 by their cost effectiveness and efficiency. That said, we follow the work in, for example^27,36^ to determine the best strategies (1-4) with the optimal cost by conducting a cost effectiveness analysis via the Incremental Cost Effectiveness Ratio (ICER). The ICER is applied to strategies *i* and *j* using the expression:

19 $$\begin{aligned} \begin{aligned} ICER(i)&=\frac{\hbox {Total Cost} (i) \hbox {}}{\text {Total infection averted} (i)}\\ ICER(j)&= \frac{\text {Total cost ({ j}) - Total cost ({ i})}}{\text {Total infection averted ({ j})-Total infection averted ({ i})}} \end{aligned}, \end{aligned}$$

where strategy *i* is the base strategy with the least utility; which in our case is the total number of new cases from period 31 to 180 days. ICER quantifies the additional cost incurred as a result of additional utility obtained for implementing a particular strategy. Table 7 gives the total infective cases and new cases, and total cost corresponding to each of the proposed strategy. We use the numerical results obtained in “Control strategies”. The period considered is between 31 and 180 days. The tables show that in terms of absolute total cost, strategy 1 has the least associated cost, followed by strategy 3, and then strategies 2 and 4. Also, we recorded the smallest total number of infective cases and new cases for strategy 1.

**Table 7:** Cost effectiveness of strategies: we introduce strategies on the 31 st day after the first record of the cases.

Strategy	Total infective cases	Total new cases	Total cost ($)
Strategy 1	411.162	28.9220	$$3.878404\times 10^5$$
Strategy 2	445.378	33.3155	$$4.184846 \times 10^5$$
Strategy 3	413.263	29.1815	$$3.896050 \times 10^5$$
Strategy 4	3937.934	508.6952	$$3.677514 \times 10^6$$

The reference period is March 16, 2020 to June 19, 2020; the values of the other model parameters are given in the Tables above. $S(0)=211{,}184{,}868,I_{NQ}(0)=1,V(0)=E(0)=I_Q(0)=I_A(0)=R(0)=0$.$A_1=\$100, A_2=A_3=\$1000,B=C_2=\$100,C_1=\$3.7$. Note that total new cases are obtained by summing new cases from day 31 to 180.

Now, to calculate ICER for each of the strategies, consider Table 8. The table ranks the total number of total new cases averted in increasing order. Notice that strategy 4 has the lowest total new cases averted, followed by strategy 2. We calculate ICER for the strategies as follow:

20 $$\begin{aligned} \begin{aligned} ICER(4)&=\frac{3.677514\times 10^6}{78313.022} = 46.959,\\ ICER(2)&=\frac{4.184846\times 10^5-3.677514\times 10^6}{78789.502-78313.022}= -6854.194,\\ ICER(3)&=\frac{3.896050\times 10^5-4.184846\times 10^5}{78792.536-78789.502}=-7159.191,\\ ICER(1)&=\frac{3.878404\times 10^5-3.896050\times 10^5}{78792.796-78792.536}=-6799.582. \end{aligned} \end{aligned}$$

The value of ICER(4) in Table 8 is larger than that of ICER(2), indicative of strategy 2 dominating strategy 4 with respective to cost effectiveness—strategy 4 is costly and less effective than strategy 2. So, we exclude strategy 4 from the competing strategies. Table 9 shows the list of recalculated ICER for strategies 2, 3, and 1. Again, comparing ICER across the competing strategies indicates that strategy 2 is expensive and ineffective to implement as it has ICER larger than that of strategy 3. We, therefore, exclude strategy 2 and recalculate ICER for strategy 3 and 1. The calculation shows that ICER for strategy 3 is bigger than that of strategy 1, indicative of ineffectiveness and more costly of strategy 3 compared to strategy 1; see Table 10. In fact, the calculation shows that $\$6799.582$ is saved for implementing strategy 1 over strategy 3. Consequently, strategy 1 (combining compliance with vaccination program and effective quarantine) is the best strategy in terms of both cost effectiveness and efficiency curbing the spread of COVID-19.

**Table 8:** Cost effectiveness in terms of total cases averted: we introduce strategies on the 31st day after the first record of the cases.

Strategy	Total new cases	Total cases averted	Total cost ($)	ICER
Strategy 4	508.6952	78,313.022	$$3.677514\times 10^6$$	46.959
Strategy 2	33.3155	78,789.502	$$4.184846\times 10^5$$	− 6854.194
Strategy 3	29.1815	78,792.536	$$3.896050\times 10^5$$	− 7159.191
Strategy 1	28.9220	78,792.796	$$3.878404 \times 10^5$$	− 6799.582

The reference period is March 16, 2020 to June 19, 2020; the values of the other model parameters are given in the Tables above. $S(0)=211{,}184{,}868,I_{NQ}(0)=1,V(0)=E(0)=I_Q(0)=I_A(0)=R(0)=0$.$A_1=\$100, A_2=A_3=\$1000,B=C_2=\$100,C_1=\$3.7$. Note that total new cases are obtained by summing new cases from day 31 to 180.

The averted cases for each strategy is calculated by subtracting the total number of new cases corresponding to the respective strategies from the total number of new cases when there are no controls. We have 78822 total number of new cases with associated social and economic cost of $\$4.094507714\times 10^8$ when no controls are in place. Strategies 1-4 are presented in increasing order of the number of infections averted

**Table 9:** Comparison between Strategies 2 and 3.

Strategy	Total new cases	Total cases averted	Total cost ($)	ICER
Strategy 2	33.3155	78,789.502	$$4.184846\times 10^5$$	5.311
Strategy 3	29.1815	78,792.536	$$3.896050\times 10^5$$	− 7159.191
Strategy 1	28.9220	78,792.796	$$3.878404 \times 10^5$$	− 6799.582

**Table 10:** Comparison between Strategies 3 and 1.

Strategy	Total new cases	Total cases averted	Total cost ($)	ICER
Strategy 3	29.1815	78,792.536	$$3.896050\times 10^5$$	4.9446
Strategy 1	28.9220	78,792.796	$$3.878404 \times 10^5$$	− 6799.582

## Conclusion

This paper discussed a COVID-19 deterministic optimal control model, of which we considered community compliance rate, vaccination coverage, and effective quarantine as control variables. These are time dependent variables for a finite time. The optimal solution path of the control system was derived, by way of establishing the existence of an optimal solution and then specifying the characteristics of the system by deriving the first order conditions. Using data set of COVID-19 daily confirmed cases, we estimated model parameters values for parameters values not currently found in the literature. We did this under two cases: One estimation procedure considered data points from March 4, 2021 to June 27, 2021, and the other, considered data points from March 15, 2020 to June 29, 2020.

The first estimation approach was to compare traditional mathematical disease models where collective efforts of individuals are not explicitly accounted for and economic epidemiological model where individual collective efforts are incorporated in the modelling framework. Given that the time series data start from March 4, 2021, it takes into account all the three control measures—compliance rate, vaccination coverage,and effectiveness of quarantine, which permits such a comparison. The traditional model assumed all model parameters as fixed, while the economic model assumed all parameters fixed except for compliance rate. The numerical simulation indicates the disparity between these two models and that behavioral influence places major role in the eradication of the disease, as such health policy should be contextualized. This means, unilaterally imposing lockdowns, face masks usage, social distancing may not yield expected results as individuals act based on available incentives. This highlights the relevance of the explicit incorporation of collective individual responses in the estimation of the disease prevalence or number of infections when designing models to inform health policy as even though the disease transmission is a population level phenomenon, decisions to prevent or treat the disease are predominantly individually made. This work further strengthens the findings in, for instance^9–11,54^, which points out inadequacies in the traditional mathematical models studied in, say^6–8^, and others for informing health policies.

The second estimation procedure is to help address the objective of the study—establishing the best intervention in terms of cost effectiveness and efficiency in curbing the spread of the disease. We addressed this problem by considering four strategies: strategy 1—combining community compliance rate with vaccination coverage and effective quarantine rate, strategy 2—combining vaccination coverage with effectiveness of quarantine, strategy 3—combining community compliance rate with vaccination coverage, and strategy 4—combining community compliance rate with effectiveness of quarantine. From the numerical results, efficiency and cost effectiveness analysis, we concluded that implementing a vaccination program coupled with effective quarantine measures and strong campaign for adhering to the non-pharmaceutical interventions such as social distancing, face mask use, frequent sanitizing of hands etc. is the effective way of eradicating the disease. We also, note that, since access to vaccine in developing countries is proving challenging, aggressive campaign on complying to non-pharmaceutical interventions and instituting effective quarantine measures can in the long run eradicate the disease.

Even though there are works^6,36,57,58^ that attempt to incorporate both governmental and individual level interventions into mathematical disease modelling framework, to the best of our knowledge these modelling frameworks fail to adequately account for the interplay of these interventions: the trade-off between, community compliance and vaccination program, community compliance and effective quarantine, and community compliance and vaccination and effective quarantine program. This paper contributes to the literature by addressing this gap by presenting an optimal control problem that accounts for individuals’ collective economic choices and available incentives, and assessment of the effectiveness of these choices in tandem with governmental interventions.

## Data Availability

The datasets used in estimating the model during the current study are available at Our World in Data https://raw.githubusercontent.com/owid/covid-19-data/master/public/data/jhu/new_cases.csv.
